# Result of Experiments in Boston with Chloroform

**Published:** 1847-12

**Authors:** W. T. G. Morton


—*Ibid.*

—

9. *Result of Experiments in Boston with Chloroform.* TO THE EDITOR,—SIR:—From your apparent interest in the success of my discovery for the alleviation of pain in surgical operations, I have ventured to lay before the readers of your Journal, a condensed account of Prof. Simpson's method of producing insensibility, also the formula for the preparation of the compound used by him, together with the result of his experiments, all of which he kindly forwarded to me by the last steamer, with an acknowledgment of the merit attached to my previous discovery of the application of sulph. ether. The result of his experiments I was resolved to test immediately, and being out of health myself, I called upon Dr. D. R. Smilie, a person of some reputation in chemical science, and invited him to my laboratory. He politely assisted me in preparing and administering the chloroform, (the name given to the new compound) also in observing its peculiar effects upon the system. As we were about to apply heat for the distilling from the mixture of the following formula—chloride of lime, one part; aqueous solution, one part; rectified spirit, one twelfth of a part—a patient opportunely arrived for the purpose of inhaling ether, and having three teeth extracted under its operation. With a little trouble she was persuaded to remain until there was a sufficient quantity of chloroform distilled for the experiment. After inhaling ether and allowing its effect to pass away, about one ounce of chloroform was put upon the sponge previously freed from the effect of sulph. ether, and administered by the usual method. In order to contract this communication as much as possible, we have purposely avoided the details of the case. The teeth were extracted without the knowledge of the patient, and we would say the effects were similar to those produced by ether.

Yours respectfully

*Saturday, Dec.* 25, 1847. W. T. G. MORTON.

—*Ibid.*

